# Multiple HPV integration mode in the cell lines based on long-reads sequencing

**DOI:** 10.3389/fmicb.2023.1294146

**Published:** 2023-12-15

**Authors:** Xiaofang Cui, Yiyan Li, Chuanpeng Zhang, Yanwei Qi, Yuhui Sun, Weiyang Li

**Affiliations:** ^1^Jining Medical University, Jining, Shandong, China; ^2^Department of Bioinformatics, School of Biological Science, Jining Medical University, Rizhao, Shandong, China; ^3^BGI-Shenzhen, Shenzhen, China; ^4^Medical Research Center, Affiliated Hospital of Jining Medical University, Jining Medical University, Jining, Shandong, China; ^5^School of Control and Computer Engineering, North China Electric Power University, Beijing, China

**Keywords:** HPV integration, nanopore sequencing, HPV16, HPV18, cervical cancer

## Abstract

**Background:**

The integration of human papillomavirus (HPV) is closely related to the occurrence of cervical cancer. However, little is known about the complete state of HPV integration into the host genome.

**Methods:**

In this study, three HPV-positive cell lines, HeLa, SiHa, and CaSki, were subjected to NANOPORE long-read sequencing to detect HPV integration. Analysis of viral integration patterns using independently developed software (HPV-TSD) yielded multiple complete integration patterns for the three HPV cell lines.

**Results:**

We found distinct differences between the integration patterns of HPV18 and HPV16. Furthermore, the integration characteristics of the viruses were significantly different, even though they all belonged to HPV16 integration. The HPV integration in the CaSki cells was relatively complex. The HPV18 integration status in HeLa cells was the dominant, whereas the percentage of integrated HPV 16 in SiHa and CaSki cells was significantly lower. In addition, the virus sequences in the HeLa cells were incomplete and existed in an integrated state. We also identified a large number of tandem repeats in HPV16 and HPV18 integration. Our study not only clarified the feasibility of high-throughput long-read sequencing in the study of HPV integration, but also explored a variety of HPV integration models, and confirmed that viral integration is an important form of HPV in cell lines.

**Conclusion:**

Elucidating HPV integration patterns will provide critical guidance for developing a detection algorithm for HPV integration, as well as the application of virus integration in clinical practice and drug research and development.

## Background

Human papillomavirus (HPV) is an icosahedral virus with a diameter of 50–60 nm. The length of the genome is about 8 K. It has a double-stranded, closed circular structure and exists in three forms, helix, open and linear. The genome contains 9 coding frames, which can be divided into 3 regions, early, late, and non-transcription regions (Morshed et al., 2014). There are more than 160 types of HPV, including 14 high-risk types that cause cervical cancer, with HPV16 and HPV18 being the most common (Rodriguez et al., 2008). With persistent HPV infection for several years, high-grade squamous epithelial lesions will occur, eventually developing into invasive cervical cancer. Integration of the HPV genome into the human genome is considered a key step in cell carcinogenesis (Banuelos-Villegas et al., 2021; Kawahara et al., 2021).

The development of second-generation sequencing provides advantages over short-read technologies and a useful tool for research on virus integration. Akagi and colleagues found a high degree of instability in front of or behind the sites of HPV integration based on whole genome sequencing, and the deletion and rearrangement of regions were commonly accompanied by HPV integration (Akagi et al., 2014). A study on the haplotype of HeLa cells examining the distance of the Myc gene from HPV integration, found that it was still influenced by integration and showed upregulated expression, indicating that HPV integration could still exert some effect when it occurred in the intergenic region (Adey et al., 2013). In 2013, Li and colleagues developed an efficient and inexpensive virus integration detection method, HIVID, based on sequence capture (Li et al., 2013). Using whole genome sequencing and HIVID, Hu and colleagues identified a large number of integration sites in HeLa and SiHa cells (Hu et al., 2015). Furthermore, they elucidated that microhomology may be an important mechanism for the initiation of HPV integration into the human genome (Hu et al., 2015). More recently, Kamal and colleagues utilized an HPV double capture method to investigate HPV integration in clinical samples from 22 patients with cervical cancer and determined that the most common integration site was in the MACROD2 gene (Kamal et al., 2021). They concluded that HPV integration can promote tumorigenesis in at least three ways: (i) destructing tumor suppressor genes and promoting oncogene expression; (ii) causing localized genomic instability, including the frequent occurrence of CNV, structural variation and other events; and (iii) leading to the high expression of E6 and E7 oncogenes. These effects can promote the rapid tumor transformation of cells (Ojesina et al., 2014; Hu et al., 2015; Rusan et al., 2015).

While methods to detect HPV integration have improved, they are based on second-generation sequencing, which mostly rely on analyzing short fragments to identify the virus integration site. There is already a lot of virus integration site information on virus integration. This information is very effective for analyzing virus integration sites and related genes, and also provides good targets for virus integration research. However, it is difficult to judge the internal structure formed by viral integration events (Li et al., 2013; Wang et al., 2013; Nguyen et al., 2018). During the study of viral integration, researchers found that viral integration events produce two endpoints, but most viral integration sites actually seen cannot be effectively paired. At the same time, information such as the insertion sequence information of virus integration, structural variation within the sequence, and both end sites formed by sequence integration cannot be effectively obtained. The lack of this information will directly lead to imperfect analysis and understanding of virus integration, which will affect subsequent study on viral integration function.

The Nanopore ONT sequencing is a new generation of single-molecule real-time electrical signal sequencing technology based on nanopores. Facilitated by motor proteins, the DNA/RNA chain combines with nanopore proteins embedded in the biological membrane. Variances in the chemical properties of DNA/RNA bases induce distinct changes in electrical signals when DNA/RNA molecule traverses the nanopore channel. By detecting these signals, the type of the corresponding base can be calculated and the real-time determination of the sequence completed.

Nanopore sequencing has the advantage of obtaining ultra-long reads. Long fragments have obvious innate advantages for solving virus integration problems. The length of sequencing reads it produces is typically 10–100 kb for long reads sequencing mode, which is enough to span the viral integration fragment and determine the two end sites formed by the integration, so that a complete and comprehensive viral integration model can be obtained. In addition, it is very effective in detecting complex regions of the genome. For example, in the highly complex HLA region, third-generation sequencing can obtain higher coverage and more accurate information (Liu and Berry, 2020; Matern et al., 2020; Liu et al., 2021). In addition, it also achieves good results in HCV typing (Riaz et al., 2021). It also can be used to detect the complex mutation of HBV genome (Zhuo et al., 2021; Li et al., 2022). Researchers utilize long reads obtained through nanopore sequencing to assemble the HPV genome in a single sample, leveraging the long-reads for a comprehensive analysis of virus integration characteristics (Brancaccio et al., 2021; Yang et al., 2021).

In mechanistic studies, nanopore long-fragment sequencing reveals that HPV can mediate translocations. In these events, HPV serves as a linker, with its two ends fusing with two different human chromosomes, resulting in a larger and more complex impact of HPV integration on the human genome (Zhou et al., 2022). Nanopore sequencing proves pivotal in unraveling the intricate details of virus integration mechanisms, showcasing its broader advantages in genomic research.

In this study, we employed third-generation NANOPORE sequencing technology to (i) systematically analyze HPV integration sequences at the integration breakpoint in three common HPV-positive cell lines, HeLa, SiHa, and CaSki, and (ii) investigate the characteristics of viral integration forming at the breakpoints. The clarification of key information on integration characteristics provides not only the basis for further research on the mechanism and function of HPV integration, but also strong support for the clinical application of HPV integration, the development of related information software as well as drug research and development.

## Materials and methods

### Cell culture

The cell lines CaSki, SiHa, and HeLa were procured from the American Type Culture Collection (ATCC). CaSki cells were nurtured in RPMI-1640, enriched with 10% fetal bovine serum (FBS; Gibco), and 100 U/mL of penicillin and streptomycin (Invitrogen). Meanwhile, SiHa and HeLa cells were cultured in Dulbecco’s modified Eagle’s medium (DMEM), supplemented with 10% FBS and 100 U/mL of penicillin and streptomycin.

### DNA extraction

The genomic DNA from HPV-positive cell lines was extracted utilizing the DNeasy Blood and Tissue Kit (Qiagen, 69506) following the manufacturer’s protocol. Subsequently, the quantification of double-stranded (ds) DNA was carried out using the NanoDrop 2000 and Qubit dsDNA HS Assay Kit (Thermo Fisher Scientific, United States).

### Detection of HPV integration

DNA samples from three HPV-positive cell lines (HeLa, SiHa, and CaSki) were purified by magnetic beads (AMPure XP Beads, BECKMAN), and the ends of DNA fragments repaired. The purified product was connected with a sequencing connector in SQK-LSK109 kit (Oxford Nanopore Technologies). In summary, DNA underwent repair using DNA Repair Mix and End repair/dA-tailing Module reagents (E7695, New England BioLabs, Ipswich, MA) and subsequent purification was carried out with AMPure XP beads (A63880, Beckman Coulter, United States). The purified DNA was washed with 70% ethanol and then eluted with nuclease-free water. Following that, sequencing adapters were affixed to the 3′ends of the fragmented DNA using Adapter Mix and Quick T4 DNA Ligase with Ligation Buffer (NEB), and a final purification step was performed using AMPure beads. Qubit (Thermo scientific) was used to quantify the constructed DNA Library accurately. After establishing the DNA library, it was added to the Flow cell (R9.4), which was then transferred to Oxford NANOPORE PromethION sequencer (Oxford Nanopore Technologies, Oxford, United Kingdom) for real-time single molecule sequencing (Deamer et al., 2016; Wuhan Benagen Tech Solutions Company Limited).

FAST5 files produced by the Nanopore sequencing device were transformed into FASTQ format through GUPPY (v3.1.5), a component of the MinKNOW software package. Subsequently, clean data were derived by excluding low-quality sequences (Qscore < 7) and linker sequences from the initial sequencing dataset, a process facilitated by Filtlong (v0.2.0). The distributions and read lengths of the data were assessed using NanoPlot.^1^ Local BLAST (NCBI-Blast-2.11.0) was used to map these sequences to the HPV genome. The virus integration sites were then detected by independently developed software HPV-TSD (Supplementary Figure S1; Meng et al., 2019).

### Analysis of structural variation

Minimap2 software (version: 2.24-r1122 parameter: -ax map-ont–MD; other parameters are the software’s default parameters) was utilized to align the clean reads of all samples with the reference genome (Li, 2018, 2021). Samtools (parameter: sort) was used to convert the mapping results from Sam (Sequence Alignment/Map) file to sorted BAM file (Binary Alignment/Map; Li et al., 2009). Samtools (version:1.16.1) was selected to count the depth and coverage (Li et al., 2009), and Sniffles (version:2.0.7) software was employed to detect structure variation and for data output (Sedlazeck et al., 2018).

## Results

### Nanopore sequencing

The library was constructed and sequenced by ONT MinION. To obtain high-quality reads, the raw reads were filtered using the Metrichore 1D base calling program and kept for further analysis if a Qscore ≥ 7 was obtained. Following the analysis of the SiHa, HeLa, and CaSki cell lines, this study yielded 637,936, 989,564, and 986,945 reads for each respective cell line. The sequencing reads span from 0 to 280 kb in length, with an average length ranging from 13.6 kb to 17.6 kb. The data quality value varies between 7 and 17, and the average Q-score falls within the range of 11 to 12.

### HPV coverage and depth in the HeLa, SiHa, and CaSki lines

**Figure 1:**
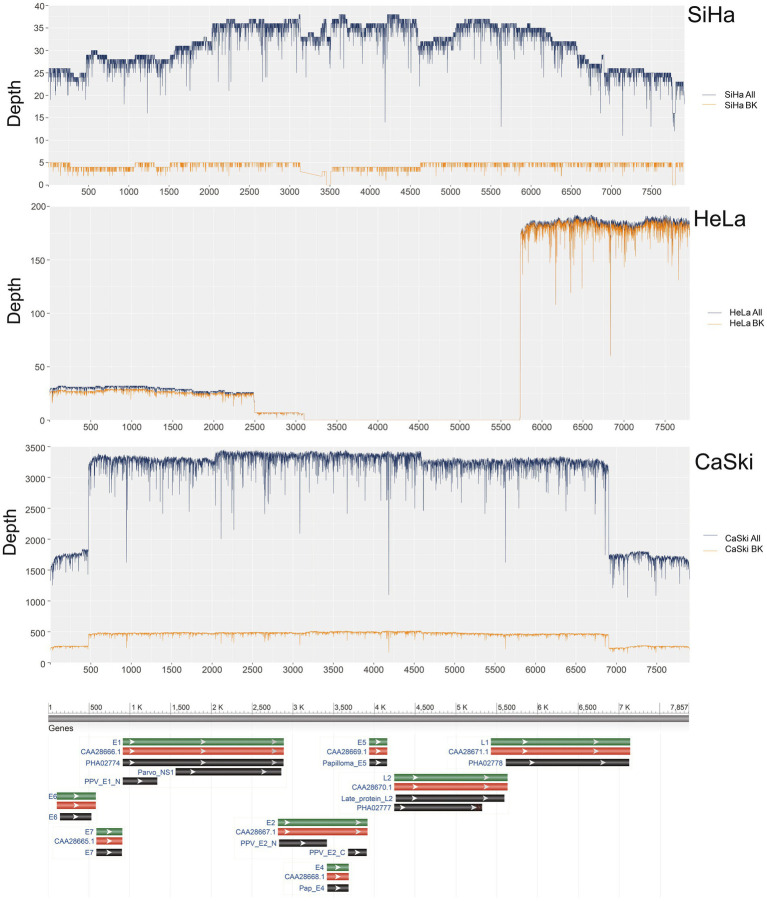
HPV coverage and depth in SiHa, HeLa, and CaSki cell lines. SiHa All (Blue Line) represented the depth of total HPV fragments; SiHa BK (Yellow Line) represented the depth of HPV fragments with human genome breakpoints; HeLa All (Blue Line) represented the depth of total HPV fragments; HeLa BK (Yellow Line) represented the depth of HPV fragments with human genome breakpoints; CaSki All (Blue Line) represented the depth of total HPV fragments; CaSki BK (Yellow Line) represented the depth of HPV fragments with human genome breakpoints.

The coverage of HPV16 in the complete sequences of the SiHa cell line was 100% with a sequence depth of 31.1x. The coverage of HPV16 in sequences with integration breakpoints reached 96.8%, with a depth of 4.3x. At least 13.8% (4.3/31.1) of the sequences existed as an integrated chromosome. The average depth of human genome was 3.3x, while the average depth of HPV16 was 9.4 times that of the human genome in the SiHa cell line. The coverage of HPV16 in the complete sequences of CaSki cell line was 100% with a depth of 2977.3×. The coverage of HPV16 in sequences with integration breakpoints was 100% with a depth of 434.3x. At least 14.6% (434.3/2977.3) of HPV sequences exist as an integrated chromosome, and the average depth of HPV is 744.3 times the 4.0x average depth of the human genome. The coverage of HPV18 in the complete sequences of the HeLa cell line was 66.5% with a depth of 58.9×. The coverage of HPV18 in the sequences with integration breakpoints was 66.5% with a depth of 57.3×. At least 97.3% of the sequences are in the integrated chromosome state, and HPV18 in HeLa cells is not intact, with the E2, E4, E5, and L2 regions missing. The average depth of the human genome is 4.9× in original sequencing data, and the average depth of the HPV18 is about 11.7 times that depth (Figure 1; Supplementary Table S1).

### HPV integration mode

**Figure 2:**
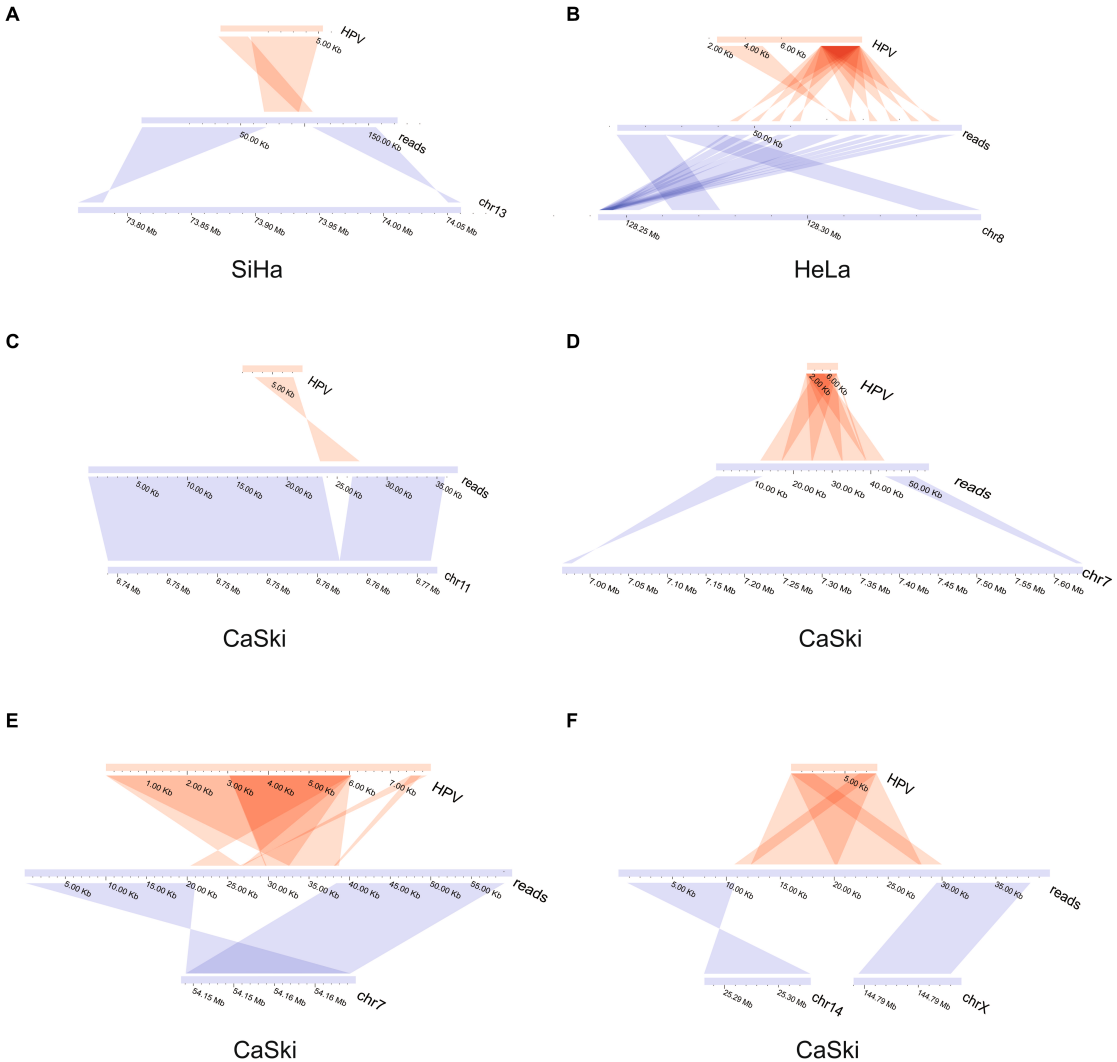
HPV integration mode in SiHa, HeLa, and CaSki cell lines. **(A)** HPV insertion sequence in the SiHa cell line; **(B)** HPV insertion sequence in the HeLa cell line; **(C)** HPV insertion sequence in CaSki cell line; **(D)** HPV insertion sequence in CaSki cell line; **(E)** HPV insertion sequence in CaSki cell line; and **(F)** HPV insertion sequence in the CaSki cell line created two breakpoints located in the different chromosomes.

The upstream and downstream region of one representative integration site was taken from each cell line to demonstrate the integration mode of the virus. The integration patterns of the representative sites in each of the three cell lines were displayed. The virus integration pattern in the SiHa cell line was dominated by partial insertion of a virus sequence (Figure 2A). For the HeLa cell line, the insertion pattern was identified through the formation of tandem repeats between the viral genome and human genome, which occurred with some regularity (Figure 2B). Some regularity means that the integrated fragment is a structural element composed of some specific regions of HPV and specific regions of the human genome, forming a continuous tandem structure on the human genome. This structure is integrated to form multiple copies on the human genome. And this phenomenon is frequently found in HPV integration. The insertion of virus integration in the CaSki cell line was relatively complex. It not only had the characteristics of multiple tandem duplications of the virus, but it also had a simple insertion pattern (Figures 2C–F; Supplementary Table S2).

**Figure 3:**
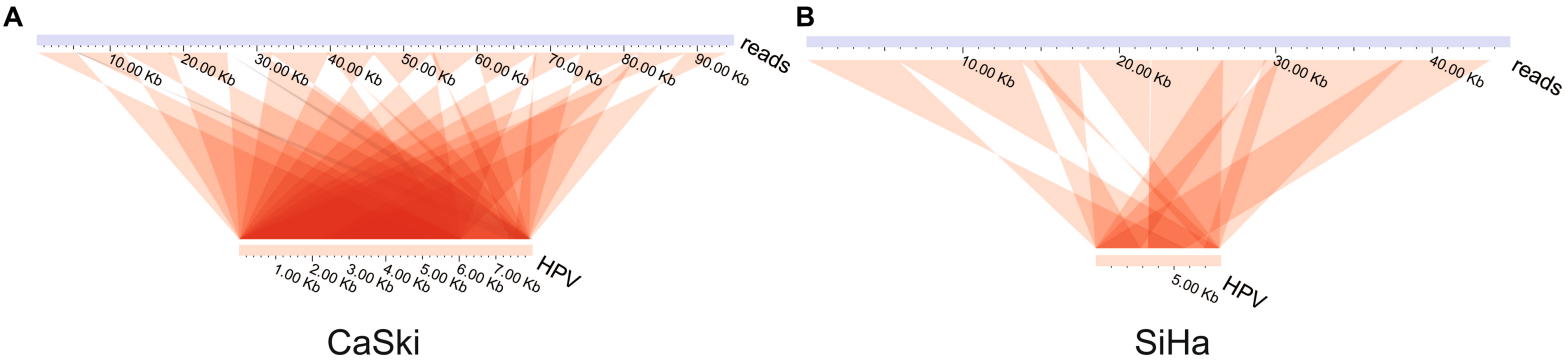
HPV genome tandem structure. HPV tandem structure in **(A)** the CaSki cell line and **(B)** the SiHa cell line.

Analysis of the ultra-long sequences of HPV in the cell lines indicated that multiple tandem duplications were formed in the ultra-long sequences of HPV. The results showed two special sequences, which are repeated multiple times in the HPV genome itself to form a tandem structure (Figures 3A,B; Supplementary Table S3). There is no viral integration site formed on them. The display of this structure shows that the existence of HPV virus integration in cells is relatively complex.

### Overall variation and integration of structural variants

**Figure 4:**
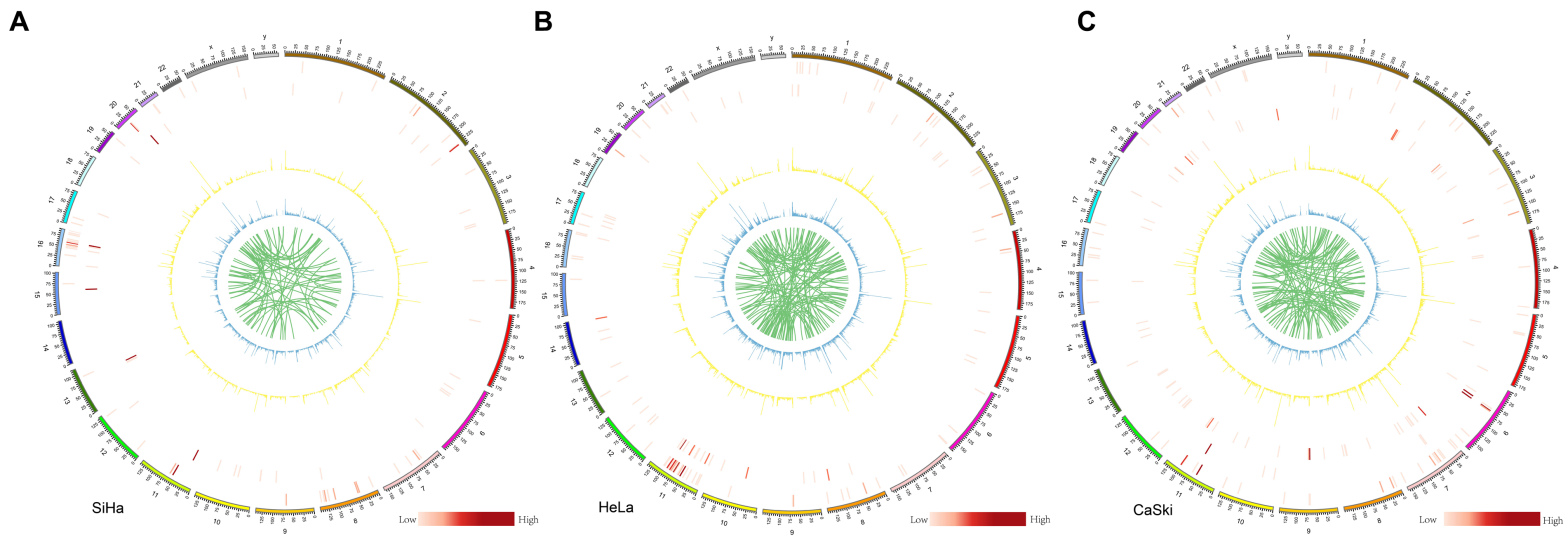
Structure variation in SiHa, HeLa, and CaSki cell lines Structure variation in **(A)** SiHa, **(B)** HeLa, and **(C)** CaSki. Cell lines from the outer ring to the inner ring: the first ring represented INV (inversion) number in each 2 M region; the second ring represented DUP (duplication) number in each 2 M region; the third ring represented HPV integration number in each 2 M region; In these three rings, the number is represented by the shade of the color. The fourth ring represented DEL (deletion) number in each 2 M region; the fifth ring represented INS (insertion) number in each 2 M region; and the sixth ring represented BND (translocation); In these three rings, the number is represented by different heights.

We systematically analyzed the five structural variants: deletion (DEL), duplication (DUP), insertion (INS), translocation (BND), and inversion (INV), as well as breakpoints (BP) in the three cell lines. In the SiHa cell line, two major integration sites were discovered. The structural variation included 95 BND types, 9,536 DEL types, 40 DUP types, 11,320 INS types, and 68 INV types (Figure 4A). In the HeLa cell line, five major integration sites were discovered. The structural variation included 137 BND types, 12,890 DEL types, 61 DUP types, 15,044 INS types, and 84 INV types (Figure 4B). For the CaSki cell line, 86 major integration sites were discovered. The structural variation included 140 BND types, 9,849 DEL types, 19 DUP types, 11,703 INS types and 73 INV types (Figure 4C; Supplementary Table S5).

### Mechanisms of HPV integration and carcinogenesis

**Figure 5:**
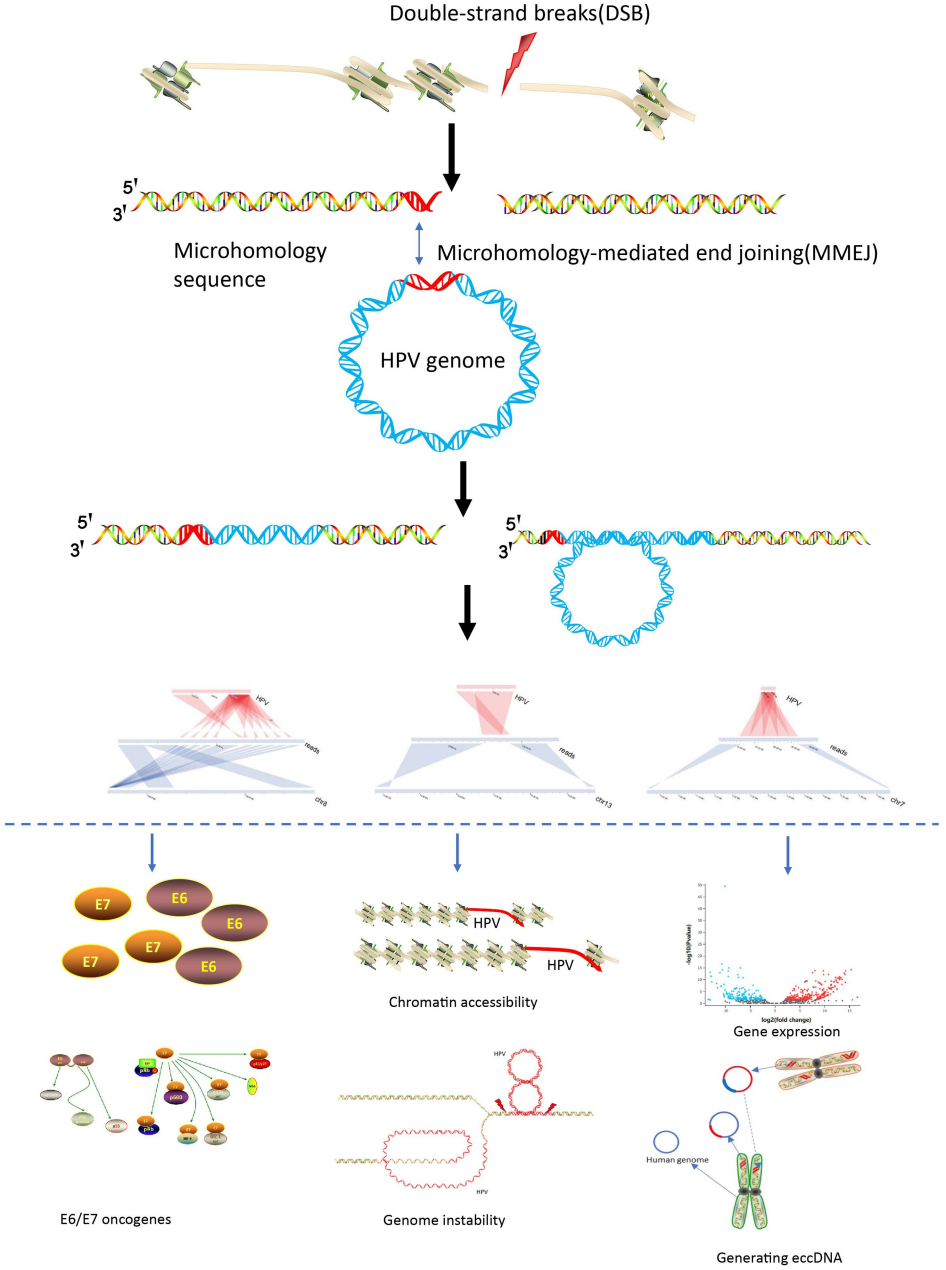
HPV integration and carcinogenesis mechanisms.

Based on the various special viral integration structures discovered in this study and previous study, we summarized the mechanisms of viral integration and carcinogenesis. The process initiates with a double-stranded breakthrough in the genome, followed by HPV genome integration based on microhomology, culminating in an integrated state through double-strand repair. After viral integration, it can cause tumorigenesis through multiple pathways: (1) Carcinogenic effects of E6 and E7 viral proteins (Matsukura et al., 1986; Thierry and Howley, 1991); (2) Viral integration destroys tumor suppressor genes and promotes high expression of oncogenes (Tang et al., 2013; Shen et al., 2017); (3) HPV viral integration leads to instability of the genome structure (Akagi et al., 2014); (4) Virus integration leads to changes in chromatin accessibility leading to widespread gene transcription abnormalities (Karimzadeh et al., 2023); (5) Through generation of oncogenic ecDNA (extrachromosomal DNA) or eccDNA (extrachromosomal circular DNA; Zhou et al., 2022; Tian et al., 2023; Figure 5).

## Discussion

These three cell lines were studied because they have different sources and are representative. HeLa is derived from cervical adenocarcinoma cell lines, SiHa is derived from cervical squamous cell carcinoma cells, and CaSki cell lines are derived from cervical cancer intestinal metastasis cells. They have their own representativeness in cervical cancer research. They are known to carry different types of HPV, HeLa (HPV18), SiHa (HPV16), and CaSki (HPV16). Furthermore, the internal structure of the integration sites of the three cell lines has not yet been fully analyzed, and the cell types in the cell lines are simple and the integration patterns are stable, making it easy to obtain comprehensive characteristics of virus integration. We discovered that the ratio of integrated HPV sequences to the overall HPV sequences in SiHa, CaSki, and HeLa cell lines was 13.8, 14.6% and 97.3%, respectively. Considering that partial sequences without breakpoints may originate from internal sequence of HPV integration events, the proportion of the integrated sequences in the three cell lines was at least as high as those mentioned above, indicating that the integration status of the HPV genome in cell lines holds significant importance.

Furthermore, the coverage of HPV18 in HeLa cells was only 66.5%. Therefore, we speculated that the virus sequences in the HeLa cell line were incomplete and existed in an integrated state. The E2, E4, E5, and L2 regions were absent in HeLa cells. The E6 and E7 regions were covered shallowly, while the coverage of L2 regions was deeper. The main function of the deletion region in HeLa cells includes the regulation of gene expression, cell cycle, and apoptosis (Graham, 2010). Therefore, it is likely that the virus integration in the HeLa cells may lead to tumorigenesis due to the loss of the above functions. The coverage in CaSki and SiHa cell lines was 100% and 96.8%, respectively, without viral genome deletion. It may suggest the carcinogenic mechanisms of HPV16 and HPV18 were different.

Our study identified HPV integration states in HeLa, SiHa, CaSki cell lines. HPV was inserted directly to form a stable insertion state and the upstream and downstream genomes were partially deleted. HPV tandem repeats were formed by the fusion of the HPV gene with the human genome. The virus integration pattern in SiHa cells was relatively simple, with partial insertion of the HPV sequence, and no complex repetition phenomenon was observed in the downstream genome. In contrast, HPV integration presented extremely complex patterns in CaSki and HeLa cell lines. The integration sites in HeLa cells often formed multiple tandem repeats based on the segment formed by fusion of the human and viral genome, indicating that viral integration can directly induce genome duplication events. We also noticed that tandem repeats existed in CaSki cells based on HPV sequences not carrying human genome sequences.

Tandem repeats of viral integration occur in both cell lines and clinical samples suggesting that this phenomenon has certain commonness and representativeness (Yang et al., 2020; Warburton et al., 2021). This can lead to a large increase in the number of virus integration sites at the same or similar sites, especially when analyzed with second-generation, short-read sequencing. It is more difficult to determine the pairing relationship between the two ends of HPV integration sites (Yang et al., 2020). The internal structure of the virus integration sequence is more complex, often involving inversion and reversion, so the role of integration is more difficult to analyze. At present, the relationship between HPV integration and disease is not well understood. We speculate that it may be closely related to the issues described above. Due to the presence of tandem repeats, the copy number variation (CNV) in localized regions will generally be formed after virus integration, directly destroying the structure and stability of the genome (Akagi et al., 2014). Some scholars believe that the formation of this structure is the result of re-integration after a certain tandem amplification of the virus integration (Balaji et al., 2021; Zhou et al., 2022; Chen et al., 2023; Dong et al., 2023). Studies have shown that HPV integration can produce ecDNA and has the function of regulating transcription. Research on tumor cells has also found that chromosomes produce a large amount of ecDNA, and then ecDNA can be integrated into the human genome again (Yi et al., 2022). This may be one of the reasons leading to the phenomenon of multiple tandem repeat integration. Other researchers consider it the result of rolling circle replication (RCR). Consequently, this dynamic process does not exclude the existence of a fusion of chromosomes (Groves and Coleman, 2018; Zhou et al., 2022). Hitherto, the mechanism of this phenomenon is still not clear.

Since the second-generation virus integration detection technology mainly relies on WGS and virus capture methods, these virus integration detection methods are mostly based on short sequence alignment to obtain chimeric reads, and then determine the gene site of virus integration based on this sequence. But the actual situation is that viral integration is very complex, and information about gene integration sites and viral genome breakpoints is only part of the information about viral integration events. Once viral integration occurs and carries multiple copies of the human genome, the information that a single integration site can represent is very limited. At the same time, it is also very important for viruses to integrate internal structural information, which is of great significance for judging protein expression, genome spatial structure, instability, etc. Therefore, the information obtained by third-generation sequencing will be of great value to the development of integrated virus detection methods. Furthermore, only by comprehensively clarifying the viral integration events can it be possible to provide more effective clinical guidance. Furthermore, once the two endpoints of the virus are integrated and the internal sequence of the virus is clearly inserted, researchers can use CRISPR technology for site-specific shearing to cut out the integrated HPV sequence from the cells, thereby achieving the goal of complete elimination. The achievement of such a viral integration clearance method will have tremendous potential in treating current viral integration diseases, including HIV and HBV. This innovative approach opens new avenues for effective treatments, marking a significant leap forward in the battle against viral integration-related illnesses. However, there are some limitations to this study. Our study uses third-generation NANOPORE sequencing technology to detect virus integration. In this study, three representative cell lines were used to study the virus integration mode. Although the three cell lines are widely used and representative in existing research, due to the complexity of HPV viral integration, the viral integration characteristics obtained through the three cell lines can only represent part of the viral integration patterns. And because Nanopore sequencing uses a non-biased sequencing method, the viral integration sequences have not been enriched, so the number of viral integration sites it detects is very limited, and low-frequency integration in cells is difficult to detect.

Clinical studies have been conducted based on integration sites and HPV typing. In terms of infection types, it was found that there are more mixed infections in the CIN stage or early stages, but once it progresses to cancer, the infection types are relatively single. Among them, virus integration in cervical exfoliated cells has been used clinically. Our early integration detection of cervical exfoliated cells has clinical guiding significance. It is proposed that positive for HPV high-risk infection and HPV integration, even with negative TCT (Thinprep Cytologic Test), the patients will be advised to carry out colposcopy examination, hence to prevent false diagnosis. For patients positive for HPV high-risk infection, but negative for HPV integration and TCT, the patients will be required to carry out short-term observation. In circumstances where patients are positive for HPV high-risk infection and integration, and biopsy of CINI, LEEP (Loop Electrosurgical Excision Procedure) or cervical conization is usually recommended. For cervical cancer and HPV integration positive patients, it is recommended to expand the scope of surgery and increase the number of post-operative examinations (Li et al., 2019). In addition, relevant researchers also found a significant association between multiple integration events and poor prognosis (Zhou et al., 2022).

However, because the comprehensive results of HPV virus integration are still unclear, most researchers at this stage are focusing on the analysis of the complete integration model of HPV virus integration, and the use of new bioinformatics and sequencing technology methods to efficiently analyze the virus integration structure. From the perspective of the perfect structure of virus integration, the pattern of HPV virus integration is more complex than researchers expected, involving various information such as HPV integration regions, multi-copy replication, and structural variation. This integration information is closely related to the dynamic process of virus integration and the instability of the human genome. It can be seen that efficient analysis of the regularity of virus integration characteristics is of great significance in its clinical application and functional research.

In summary, this study revealed the detail of HPV integration characteristics. Defining these characteristics is beneficial to further guide investigations of HPV integration, promote the development of new bioinformatics methods, and effectively provide precise targets for the edition and deletion of HPV sequences in the genome.

## Data availability statement

The data presented in the study are deposited in the NCBI repository, accession number PRJNA887187.

## Ethics statement

Ethical approval was not required for the studies on humans in accordance with the local legislation and institutional requirements because only commercially available established cell lines were used.

## Author contributions

XC: Conceptualization, Writing – original draft, Investigation. YL: Formal analysis, Methodology, Software, Writing – review & editing. CZ: Formal analysis, Methodology, Writing – review & editing. YQ: Formal analysis, Methodology, Writing – review & editing. YS: Methodology, Writing – review & editing. WL: Conceptualization, Writing – original draft.

## Footnotes

^1^https://github.com/wdecoster/NanoPlot
