# Supplementary figures and images for: Multiple HPV integration mode in the cell lines based on long-reads sequencing
Source: Front Microbiol. 2023 Dec 15;14:1294146. doi: 10.3389/fmicb.2023.1294146 (PMC10758443; doi:10.3389/fmicb.2023.1294146)

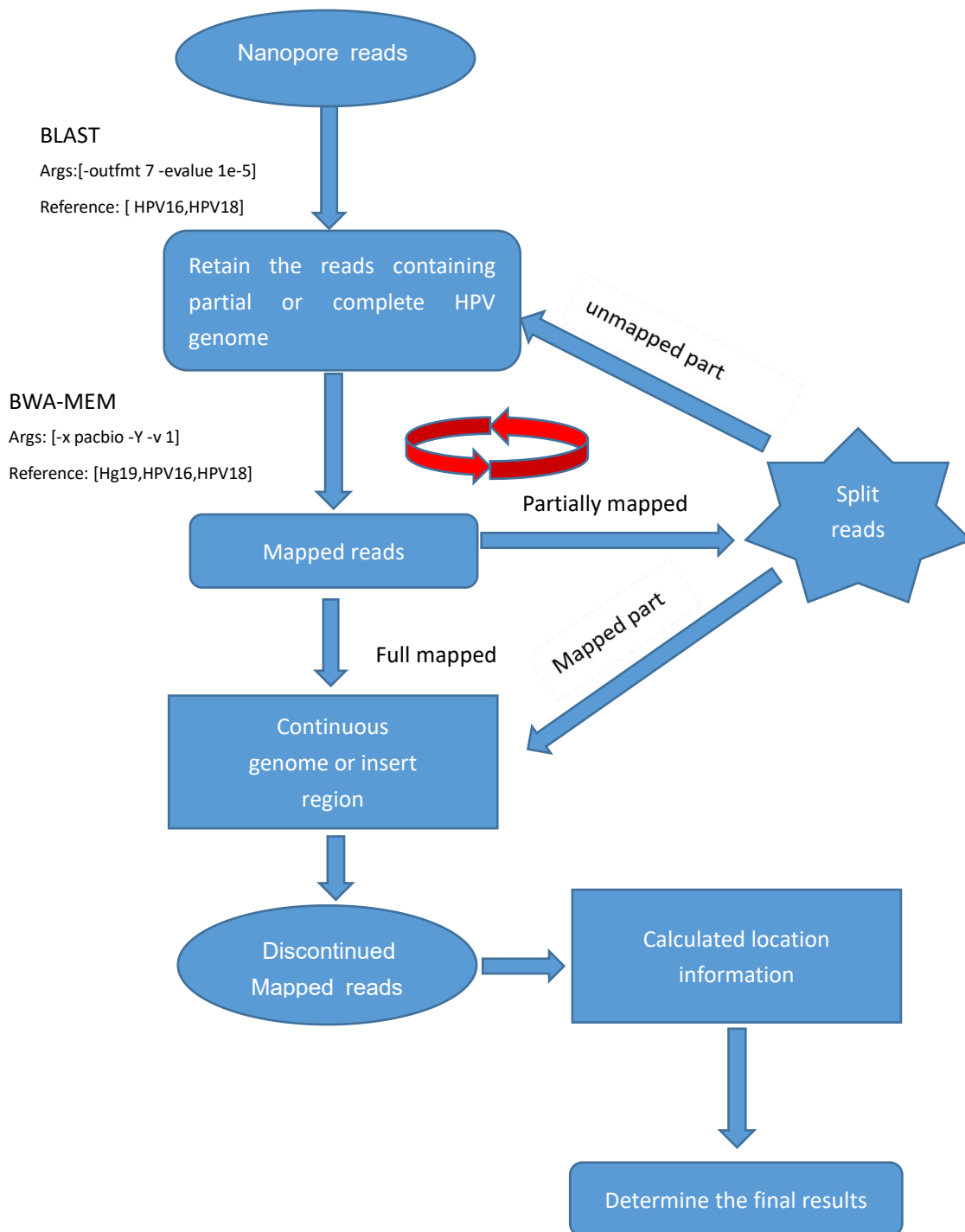

Supplement: Supplementary file 6 [file Image_1.PDF]
